# Where are the cruise ships? mobility and immobility of cruises under COVID-19

**DOI:** 10.1371/journal.pone.0303216

**Published:** 2024-11-06

**Authors:** Xumao Li

**Affiliations:** Transport Planning and Research Institute, Ministry of Transport, Beijing, China; Fiji National University, FIJI

## Abstract

The aim of this study is to reveal the spatial-temporal characteristics and influencing factors of the static and dynamic distribution of global cruise ships, against the backdrop of the COVID-19 pandemic, which has transformed the global cruise network from "lines" to "points". This study collected trajectory data for 292 cruise ships and data for 789 cruise ports worldwide from March 2020 to July 2021. Based on the relationship between port and navigation, port management rights research, and port geography theory, we analyzed the spatial distribution and spatiotemporal migration of cruise ships with ArcGIS tools. It was found that, compared with normal times, the distribution of cruise ships in regional markets, countries, and ports showed stronger spatial agglomeration characteristics and formed four types, which were mainly influenced by differences in cruise operators’ fleet scale, positioning, and itinerary. With the improvement of the epidemic, cruise ships trended to gather at the cruise home port. During the COVID-19 pandemic, there was an obvious separation between the epidemic prevention country, flag country, and operator country. Operators were inclined to berth their cruise ships in the countries where cruise ships were registered, countries of operators, and ports with high integration. Rather than simply emphasizing the static state of the cruise shipping network, the global cruise geography under the COVID-19 pandemic reflects the right relationship between ports, cruise ships, and companies. This study provides a methodological framework for analyzing the cruise shipping network at the port level and has practical implications for micro-interpretation of the dotted cruise shipping network during the COVID-19 pandemic.

## Introduction

The spread of COVID-19 has had a serious impact on the cruise industry [1]. Since February 2020, when cases of COVID-19 were first reported aboard the Diamond Princess, which sailed from its home port in Yokohama, Japan, most cruise ships sailing in the Asian region have been refused to dock by port authorities, and cruise lines have been forced to adjust regional itineraries [2].

Most cruise ships, including Holland America Line, Princess Cruises, Dream Cruises, and others, received a denial of call notices from ports in Asian countries or regions such as Japan, Thailand, and Singapore in February 2020 [3]. Norwegian Cruise Line, Celebrity Cruises, and Royal Caribbean Cruises have adjusted their Asian deployments, canceling some regional itineraries and issuing deployment announcements that would be followed by replacement cruise schedules [4].

With the global pandemic of COVID-19, unprecedented global travel restrictions, closing borders, and more cruises canceled in Asia. Besides, cruise ships outside Asia have also been severely affected. Most cruise lines have announced a change to the company’s cancellation policy, citing escalating worldwide concerns over the spread of COVID-19 [3]. On 14 March 2020, members of the Cruise Line International Association (CLIA) voluntarily suspended their cruise ship operation due to the COVID-19 pandemic, and on 12 April 2020 a No Sail Order was issued by the Centers for Disease Control and Prevention (CDC) suspended all cruise operations until 30 September 2020 [5]. As shown in Table 1, by the end of March 2020, cruise lines including Carnival, Royal Caribbean, and Norwegian Cruise have announced the suspension of all their cruise ships.

**Table 1:** Outage schedule of some cruise lines.

Cruise line	outage time	Cruise line	outage time
**Princess Cruises**	Mar12, 2020	Celestyal	Mar12, 2020
**Viking**	Mar12, 2020	Windstar	Mar13, 2020
**Fred. Olsen**	Mar13, 2020	Disney Cruise	Mar13, 2020
**Norwegian Cruise**	Mar13, 2020	AIDA	Mar13, 2020
**Royal Caribbean**	Mar13, 2020	Costa	Mar13, 2020
**MSC**	Mar13, 2020	CMV	Mar13, 2020
**Carnival Cruise**	Mar14, 2020	American Queen Steamboat	Mar14, 2020
**Cunard**	Mar14, 2020	Holland America	Mar14, 2020
**Seabourn**	Mar14, 2020	Hurtigruten	Mar18, 2020
**Bahamas Paradise**	Mar15, 2020		

Note: Information from Cruise Industry News, collated by the authors.

Cruise ships are facing the problem of where to go, which makes them homeless and become stray cruises for a while, because of the global travel ban and the closed cruise ports [1]. Existing studies focus more on mobile cruise ships because mobility is one of the key attributes of cruise ships [6]. There is no doubt that the outbreak of COVID-19 is a catastrophic event for the cruise industry. Some cruise lines are facing the risk of bankruptcy. However, it also provides an opportunity to observe the spatial relationship between ports and cruise ships in a relatively static state during a particular period.

This paper focuses on the geographical distribution of cruise ships in the context of cruise ships being forced to stop sailing under the influence of COVID-19 and tries to explain the internal reasons for its formation. We selected two key nodes in COVID-19 to study the spatial distribution of cruise ships and compared changes in their locations. It should be noted that the study samples in this paper are all "non-serviced cruise ships", where there were no cruise passengers on board, but there was the necessary staff. In addition, during the epidemic period, a few cruise ships attempted to resume their voyage, such as Costa Cruise Lines and MSC, which resumed their voyage with no destination itineraries or itineraries composed of ports in a single country [1]. To avoid the impact of this situation on the conclusion of the study, we identified these cruise ships and eliminated them.

This paper is structured as follows. Following on from the Introduction. Section 2 presents a literature review that covers the advances in cruise research under COVID-19. Section 3 introduces the samples and data. Section 4 describes the spatial distribution of cruise ships in the global scope. Section 5 analyzes the displacement variation and formation reason of cruise ships at different time nodes. Section 6 analyzes the difference in cruise ships’ choice of port of call at the enterprise scale, then reveals the reasons for such spatial differences deeply. Concluding remarks are given in the final section.

## Literature review

### Cruise mobility

Cruises are not a simple form of travel or an ordinary tourist product. They are one of the current emblematic manifestations of transnational capitalist tourism [7]. Cruises involve interrelations and Spatio-temporal repercussions on multiple levels [8]. Cruises promote the creation of relational spaces around port areas in the periphery, but also throughout host territories’ regional and national space [9]. However, the fundamental distinction between the mass cruise tourism industry and the exclusively land-based tourism industry is the former’s extreme mobility [10]. Mobility is generally structured along a center/periphery axis, and more generally operates on a neocolonial dynamic, it would be advisable to refocus its control within the spaces of tourist destinations, allowing these to determine their territorial development. The mobility from which the industry benefits thanks to its floating infrastructure allows it to easily break the geographic ties that link it to the destinations with which it comes in contact [11]. Global mobility is one of the fundamental conditions for the viability of the cruise. For the cruise industry, mobility is irreversible: growth is rooted in the notion of space production and spatially fixing capital [12], the latter of which is the basis of the production of the tourism space necessary for the cruise industry’s survival. Mobility is necessary for the cruise industry, but it can also be a weakness [8]. The COVID-19 pandemic put an abrupt end to the mobility on which the cruise relies. Destinations began to refuse to receive stopover vessels that were still at sea when the virus began to spread at an alarming rate, and most Western countries closed their borders to nonessential transit [7]. This was exacerbated by socio-territorial exclusions in special circumstances, which led to the reduction of the power scope and power space of the cruise party.

Mobility is an important criterion for cruise destinations, besides being an economic resource for the cruise ship. Mobility will also depend on regional or territorial transport infrastructure [13]. To some extent, the construction of cruise terminals has increased the docking options of cruise ships. In this sense, it has increased the mobility of cruise ships. The first approaches to cruise passenger mobility were developed through observational methods [14]. Recently, the spatial-temporal behavior of cruise passengers at their destination was studied by tracking technologies and big data to solve the congestion effect [15,16].

### Cruise and COVID-19

Due to the mobility of the cruise ship, the closure of the ship space, and the image of cruise ships has been transformed from “Floating luxury hotels/resorts” into “Floating Petri dishes”, during the COVID-19 epidemic. After the outbreak of COVID-19 on cruise ships, many scholars adopted methods and means such as investigation, prediction, evaluation, and simulation to analyze and study the characteristics, transmission routes, and infection rate of COVID-19 cases on cruise ships. Nishiura H [17] and Zhang et al., [18] were Concerned about the incidence of COVID-19 on cruise ships and believed that movement restriction was necessary which could reduce the number of infected people. Mizumoto et al., [19] Liu et al., [20] Hoshino et al., [21] and Huang et al., [22] highlight the high transmissibility of COVID-19 in confined settings of cruise ships. Therefore, strictly dividing epidemic control and vaccination, and tracing cruise passengers and contacts using big data after the cruise ship docks are essential [23–25]. Besides, Liu et al., [20] comprehensively provided solutions for the prevention and control of the cruise ship epidemic with short-term response measures and long-term mechanism construction.

Cruise ships that are canceled and have no port to berth have a serious impact on the physical and mental health of those on board. The COVID-19 pandemic in the cruise industry has managed to erase the feeling of joy from cruise ship employees who were stuck at sea [26], associated hospital workers on the cruise ship also experienced psychological distress [27]. Therefore, it should not be ignored to help cruise passengers and staff with emotional changes such as psychological anxiety and health concerns [28–31].

Through the impact of the COVID-19 pandemic on the cruise industry, some scholars have provided insights from a critical perspective for cruise development in the post-pandemic period. Pallis and Papachristou [6] pointed out the importance of port governance models and the direction of future development. Brewster et al., [32] commented on the US government and cruise ship industry’s response to the COVID-19 pandemic, and recommendations for the cruise ship sector going forward. Renaud [8] analyzed the advantages and disadvantages of the mass cruise tourism industry through the outbreak of the epidemic, thoughted that mobility of the mass cruises was a neocolonial dynamic, and proposed future development alternatives aligned with deglobalization and degrowth of the industry. Gössling et al., [33] explored how the pandemic may change society, the economy, and tourism, and questioned the volume growth tourism model advocated by UNWTO, ICAO, CLIA, WTTC, and other tourism organizations.

In summary, based on the impact of COVID-19, many pieces of research have focused on the people involved in the cruise, and the cruise industry. These studies are conducted at the level of people or industries, but rarely from the perspective of cruise ships. Cruise ships and ports are a pair of inseparable objects. Therefore, under the impact of the pandemic, cruise ships cannot be analyzed in isolation from ports. Normally, there is a mutually selective spatial relationship between the cruise port and cruise ship, and the cruise ship owns greater choice. Because the industry benefits from the mobility of its floating infrastructure, it is easy to break the geographical ties that bind it to ports. If the conditions under which their activities are deployed do not meet their commercial expectations, it is possible to relocate else ports [11]. However, affected by COVID-19, the option of cruise ships is weakened, while the port increases. This leads to a change in the relationship between ports and cruise ships. The impact of this change on the spatial distribution pattern of cruise ships, as well as the underlying reasons, are worthy of further study.

## Data and description

**Fig 1:**
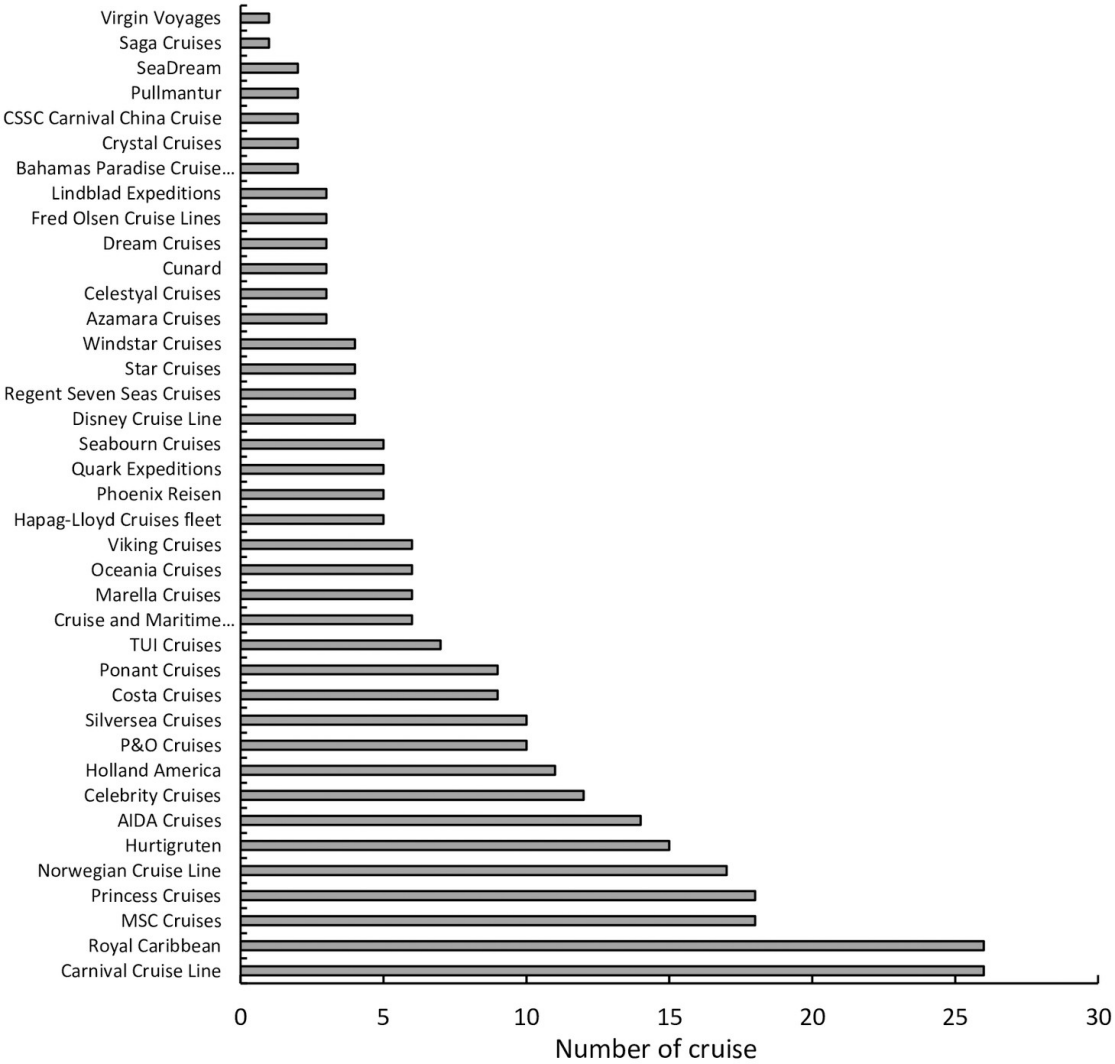
Number of cruise ships from 36 operators.

The sample data types of the study include the archival data of the cruise ship and the trajectory data of the cruise ship. As shown in Fig 1, the archive data includes 292 cruise ships which all exceed 10,000 tons and belong to 36 cruise operators. The trajectory data includes the cruise ship’s spatial position and movement direction from March 2020 to July 2021. which mainly comes from Lloyd’s Register of Shipping, an authoritative classification society.

**Fig 2:**
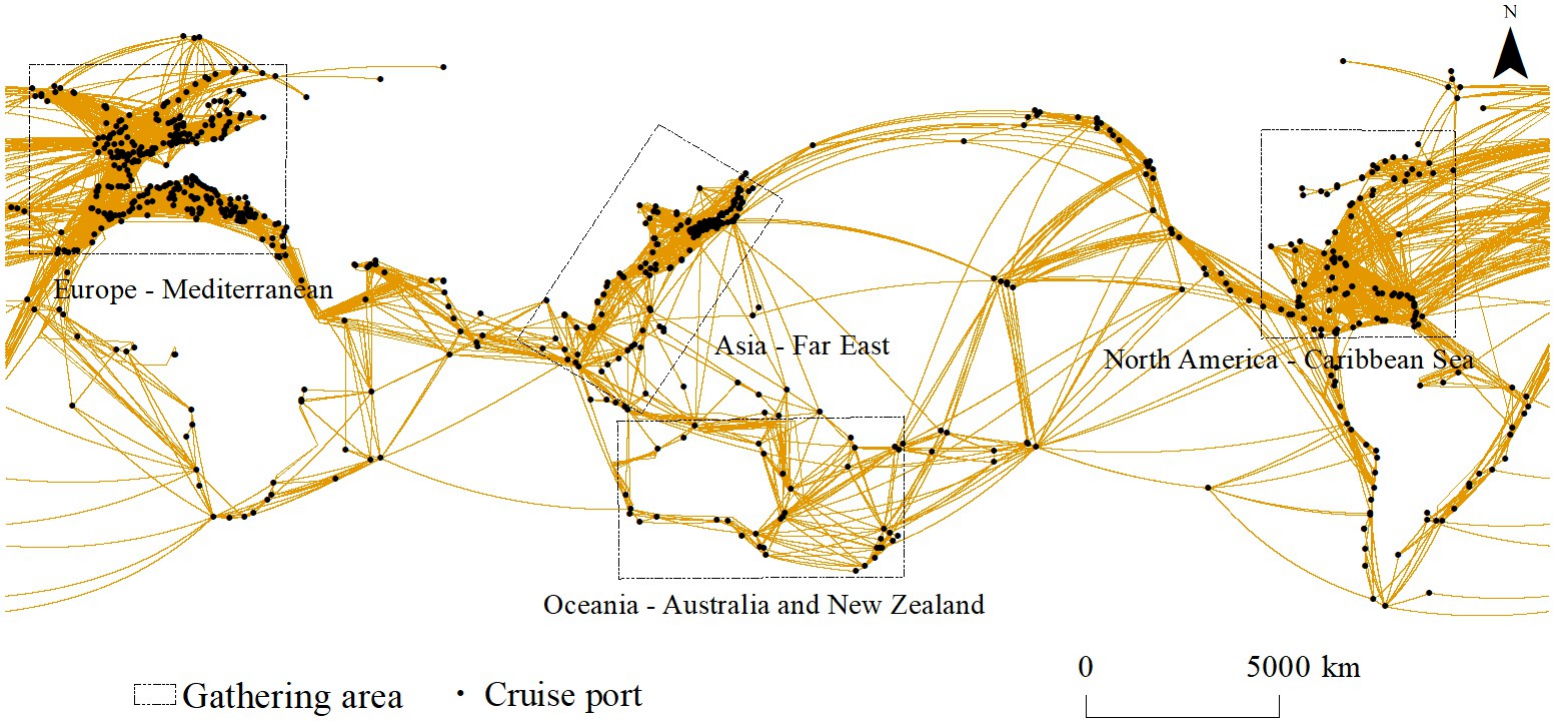
Global cruise shipping network and gathering area in 2019.

It should be noted that cruise ships could berth at more than 800 cruise ports in most coastal countries or regions around the world, forming a shipping network pattern covering globalization, before the COVID-19 pandemic in 2019 [1]. At the same time, due to the development differences between regional markets, including the level of regional economic development, port infrastructure conditions, the size of the tourist source market, natural geographical seasonality factors, etc. cruise shipping networks also showed obvious spatial clustering characteristics, especially the North America—Caribbean region, the Europe—Mediterranean region, the Asia—Far East region and the Oceania—Australia—New Zealand region (Fig 2).

Most cruise ports refused the berth of the cruise ship, and cruise lines around the world could not operate intra-regional and inter-regional cruise itineraries, which led to fundamental changes in cruise shipping networks and showing a static spatial distribution pattern of dots, during the COVID-19 pandemic. The stationary state of the cruise ship is relative. To ensure the normal operation of the ship, cruise operators need to dock the ship at the port for maintenance, personnel rotation, etc., and also need to start the ship on irregular sea voyages to keep running properly. In addition, considering the arrival of the possible resumption period, the distribution of cruise ships needs to be adjusted in advance. Also, some cruise ships have carried out special missions during the COVID-19 pandemic, as the Carnival Paradise was recently used to evacuate residents of St. Vincent after the imminent eruption of the La Soufriere Volcan after its cruises were suspended. For these cruise ships, this study is eliminated through data cleaning to ensure the reliability of data.

## A framework for analyzing cruise shipping networks under COVID-19

**Fig 3:**
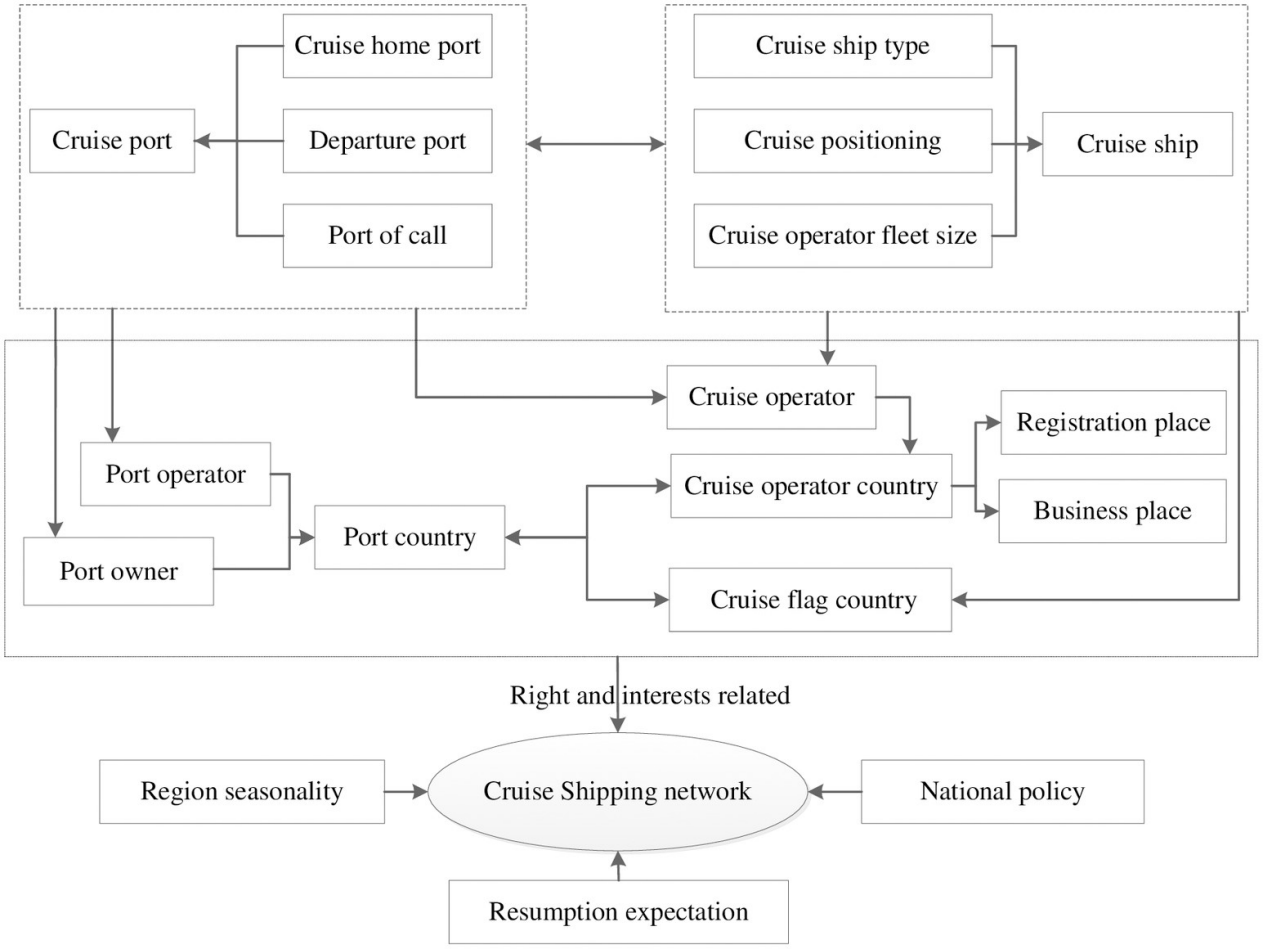
An analytical framework based on rights and interests.

As shown in Fig 3, for the analysis of the special spatial pattern of cruise shipping networks under the influence of COVID-19, this paper proposes a new theoretical analysis framework, which mainly starts from the two factors of port and cruise. By tracing the ownership of these two factors to the stakeholders, we can analyze the rights and stakeholder relationships of different stakeholders to reveal the internal logic and mechanism of the formation and development of the global cruise shipping network.

## Immobility of cruises

### Static agglomeration at different scales

1. Cruise ships gather significantly in the middle and low latitude, mainly in the northern hemisphere, especially in the range of 20°N~70°N, 0°~50°E, 0°~50°N, 50°W~120°W. The number of cruise ships in this range accounts for 98.6%.
2. The agglomeration and distribution characteristics of cruise ships in Europe, America, and Asia are significant. In terms of intercontinental scale, cruise ships are distributed in 5 continents except for Oceania and Antarctica, including South America West, Tahiti, Caribbean, North America West, Hawaii, Northern Europe, Western Europe, Southwest Europe, Mediterranean, Southeast Asia, South Asia, Central West Asia, Australia East, Africa, a total of 14 regions. However, cruise ships distributed in Europe, North America, and Asia accounted for more than 97% of the total cruise ships, especially in the Mediterranean, Caribbean, Southeast Asia, and Western Europe, the four regions gather 74.5% of the cruise ships. These four regions are the main passenger market areas for cruise activities in normal times [34]. Therefore, it can be concluded that the outbreak of COVID-19 has not fundamentally changed the regional agglomeration characteristics of global cruise ships, despite significant changes in cruise shipping networks.
3. The agglomeration distribution of cruise ships in European countries is obvious. In terms of the countries, cruise ships dock in 38 countries and regions, mainly in Italy, the United Kingdom, the United States, France, Greece, Singapore, Norway, Spain, and Germany. These countries have more than 200 cruise ships, accounting for 70% of the total. Excluding Singapore and the United States, the remaining 7 countries are all European, particularly Italy, the United Kingdom, and France.

### Choice of cruise functional ports

Cruise ports can be divided into two categories, cruise home port or departure port and the port of call. Only about 90 ports have been chosen by cruise ships to dock in during the COVID-19 pandemic. Compared with normal times, the number of ports has decreased by 90%. From the perspective of port function, the ports usually have the function of departure, especially some home ports. In the early days of the COVID-19 pandemic, 65.4% of 292 cruise ships stopped at the cruise home port or departure port. Until July 2021, that proportion had increased to 79%. This reflects the trend for cruise ships to further converge to the departure port or home port during the pandemic. These ports are the Miami cruise port, Singapore cruise port, Civitavecchia cruise port, Marseille cruise port, Los Angeles cruise port, etc. Each of these ports docks more than 10 cruise ships and usually has a more complete infrastructure and ship maintenance conditions.

### Distribution pattern of cruises at the enterprise level

**Fig 4:**
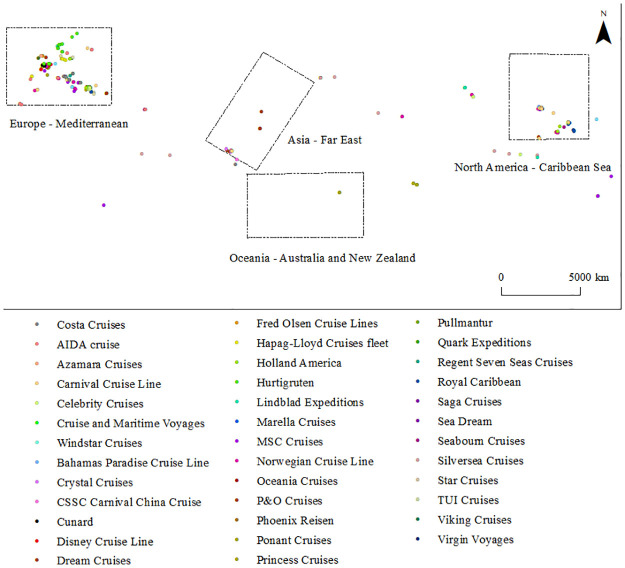
Spatial distribution pattern of 36 operators’ cruise ships in 2020.

**Fig 5:**
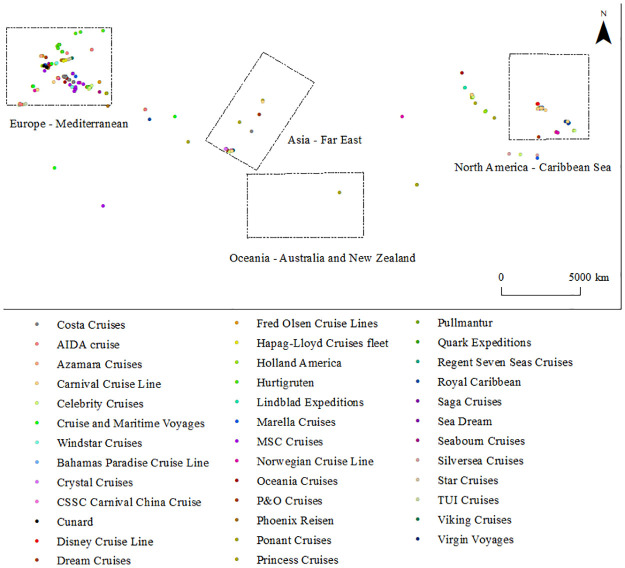
Spatial distribution pattern of 36 operators’ cruise ships in 2021.

The operator acts as the direct organizer of the cruise ship and determines the choice of space for the cruise network. The study compares ship deployment patterns for all cruise operators in 2020 and 2021. The differences in fleet size, itinerary positioning, and other aspects among the 36 cruise operators led to diversified ship space deployment during the COVID-19 pandemic (Figs 4 and 5). Generally speaking, the ships of large cruise operators show the characteristics of a scattered layout. With the reduction of the fleet size of cruise operators, the docking deployment of cruise ships presents a cluster layout area.

#### (1) Single regional type

Thirteen operators, including Cunard, Azamara Cruises, and Dream Cruises, have chosen to concentrate all their vessels in a single area, and their clustering characteristics are significant. These operators’ fleets are small, averaging 2 ships per company. Among them, Cunard, Azamara Cruises, Cruise and Maritime Voyages, and Saga Cruises choose to dock all their ships in western European regional waters, and all choose British ports, mainly Southampton Port and Grianaig Port. Dream Cruises, Crystal Cruises, and CSSC Carnival China Cruise choose to concentrate their Cruise operations in Southeast Asian waters, particularly the ports of Singapore and Klang. Celestyal Cruises, Aegean, Pullmantur, and Virgin Voyages concentrate their cruise ships in the Mediterranean. The Bahamas Paradise Cruise Line chooses the Port of Miami on the Florida Peninsula and the surrounding waters.

#### (2) Two-region type

Thirteen operators, including Regent Seven Seas Cruises, Phoenix Reisen, and Quark Expeditions, have their ships spread across two regions. Except for Lindblad Expeditions, which has only three cruise ships, each of the other 12 operators has more than four ships, while Ponant Cruises and Costa Cruises have fleets of nine. Three operators, including Marella Cruises, Ponant Cruises, and Windstar Cruises, have chosen to dock their ships in the Mediterranean and Western Europe. In contrast, Seabourn Cruises and Regent Seven Seas Cruises choose the Caribbean and Mediterranean. More than 80% cruise ships of Costa Cruises are distributed in the Mediterranean, especially in Italian ports and nearby waters. Four ships of Star Cruises are concentrated in Southeast Asia, especially Singapore, and only one is in Western Europe. Four ships of Disney Cruise Line are in Western Europe and the Caribbean, especially in ports in the United Kingdom and the Florida peninsula. Phoenix Reisen has four ships in Western Europe and only one in the Red Sea region. Quark Expeditions has four ships in southwest Europe and only one in the Mediterranean. Lindblad Expeditions chooses Northern Europe and South America.

#### (3) Three regional types

These operators include Viking Cruises, Oceania Cruises, TUI Cruises, and Hurtigruten. Their cruise distribution shows a significant European orientation. The first three operators each have 6 to 7 ships. Oceania Cruises has 4 ships parked in the Mediterranean Sea, while the other two operators distribute their ships in ports in Western Europe, Northern Europe, and the Mediterranean region. However, Hurtigruten, the largest adventure cruise operator, has 10 ships, but 75% of its ships are in Norwegian coastal ports and nearby waters.

#### (4) Multi-region type

Ten operators, including Norwegian Cruise Line, MSC Cruises, and Princess Cruises, have an average number of 16 Cruise ships, among which, Royal Caribbean and Carnival Cruise Line each have 26 ships. These cruise operators spread their ships across four or more regions, covering almost every major market in the world, except for Alaska, Australia, and New Zealand, and the Arctic and Antarctic regions. 80% of cruise ships of Norwegian Cruise Line and Celebrity Cruises are concentrated in the Mediterranean Sea and the Caribbean Sea. 50% of cruise ships of P&O Cruises are in western European waters, particularly in British ports. 60% of cruise ships of Royal Caribbean are in the Caribbean and around the Florida peninsula. Carnival Cruise Line has 16 ships off the east coast of Florida, particularly near the port of Miami. 70% of MSC Cruises ships are in the Mediterranean Sea. In addition, Silversea Cruises, Princess Cruises, AIDA Cruises, and Holland America distribute cruise ships relatively evenly in the Caribbean, Mediterranean, Southeast Asia, and Western Europe.

### Agglomeration pattern of cruises in port

**Fig 6:**
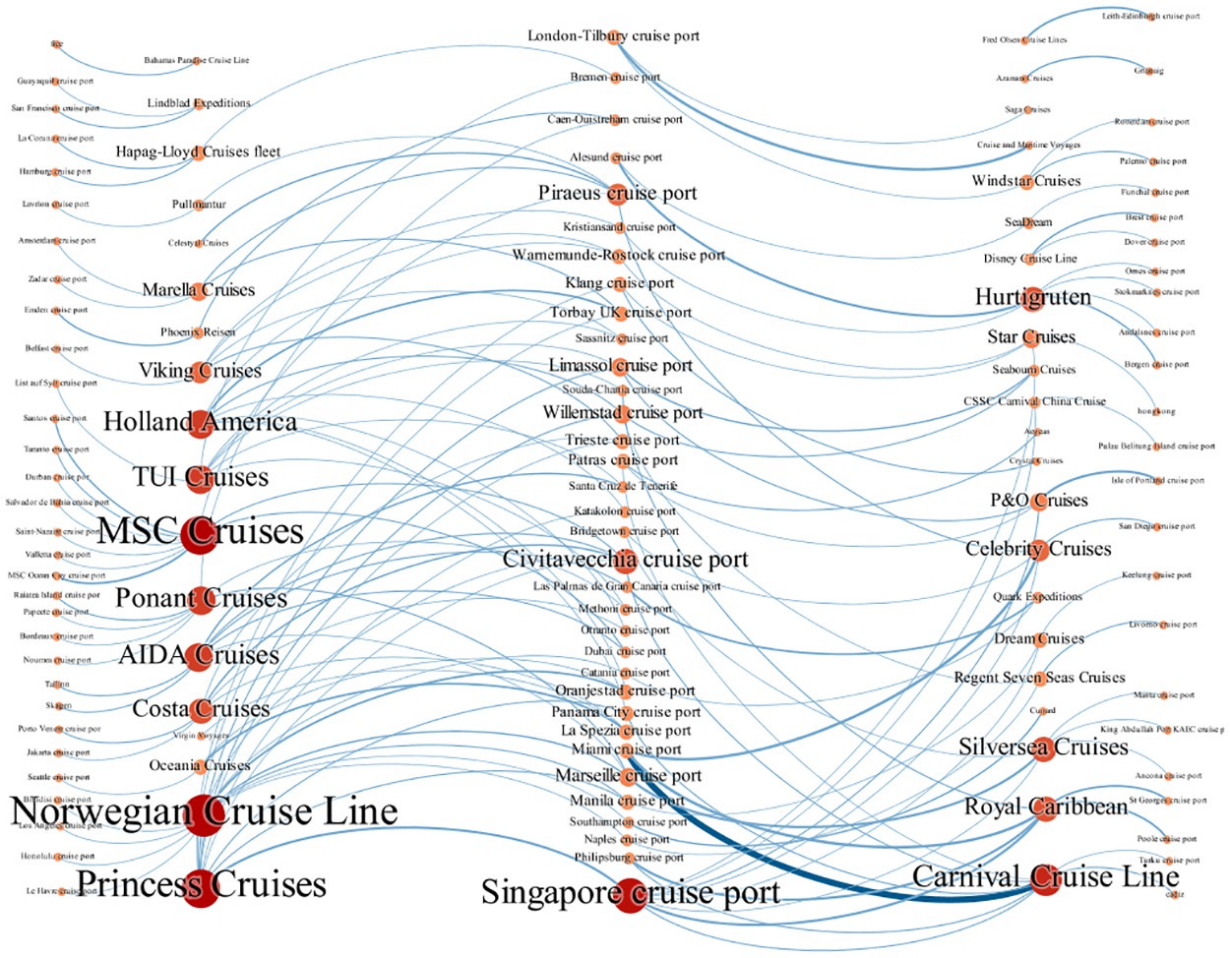
Structure of 36 cruise operators docking in ports.

1. As shown in Fig 6, during the COVID-19 pandemic, Dover cruise port, Emden cruise port, and other 57 ports hosted 96 cruise ships that belonged to 27 operators. Each of these ports has only one operator’s ship docking. On the contrary, 34 ports, such as Singapore port, Miami port, and Naples port, each have at least 2 operators coming to dock, serving a total of 196 ships from 31 operators. It can be seen that under the impact of COVID-19, the choice of ports by operators has led to the differentiation of some dedicated ports and public ports. In terms of port function, more than 64% of public ports belong to departure ports or home ports, while only 12 ports belong to ports of call. More than 57% of dedicated ports are ports of call, and only 24 are departure ports or home ports.
2. Due to the difference in fleet size and deployment strategy, the distribution of operators in ports mainly presents three types of differences. Nine operators, including Azamara Cruises, Bahamas Paradise Cruise Line, and Fred Olsen Cruise Lines, each choose to dock all their ships in one port. Cruise and Maritime Voyages concentrates its six cruise ships in the London-Tilbury Cruise departure port, while Azamara Cruises concentrates its three ships in the Grianaig port of call. Twenty operators, such as AIDA Cruises, Costa Cruises, and others, chose to spread their ships relatively evenly across different ports. Among them, TUI Cruises has seven ships at seven ports, including Bremen cruise port and Limassol cruise port, etc. Star Cruises has four ships docking at four ports including Klang cruise port and Singapore cruise port. MSC Cruises has 18 ships docking at 10 ports including Civitavecchia cruise port and Dubai cruise port. Meanwhile, 10 operators, including Carnival Cruise Line, Celebrity Cruises, and Hurtigruten, scattered their ships to different ports but concentrated most of their ships in a few ports. Carnival Cruise Line has 26 ships scattered across eight ports, but 14 ships are concentrated in the Miami cruise port. Ten ships of P&O Cruises are spread across four ports, but 5 are concentrated in the Isle of Portland Cruise Port. Eighteen ships of Princess Cruises are distributed in 10 ports, but 5 are concentrated in the Singapore cruise port.

## Mobility of cruises

### The regional characteristics of cruises migration

**Fig 7:**
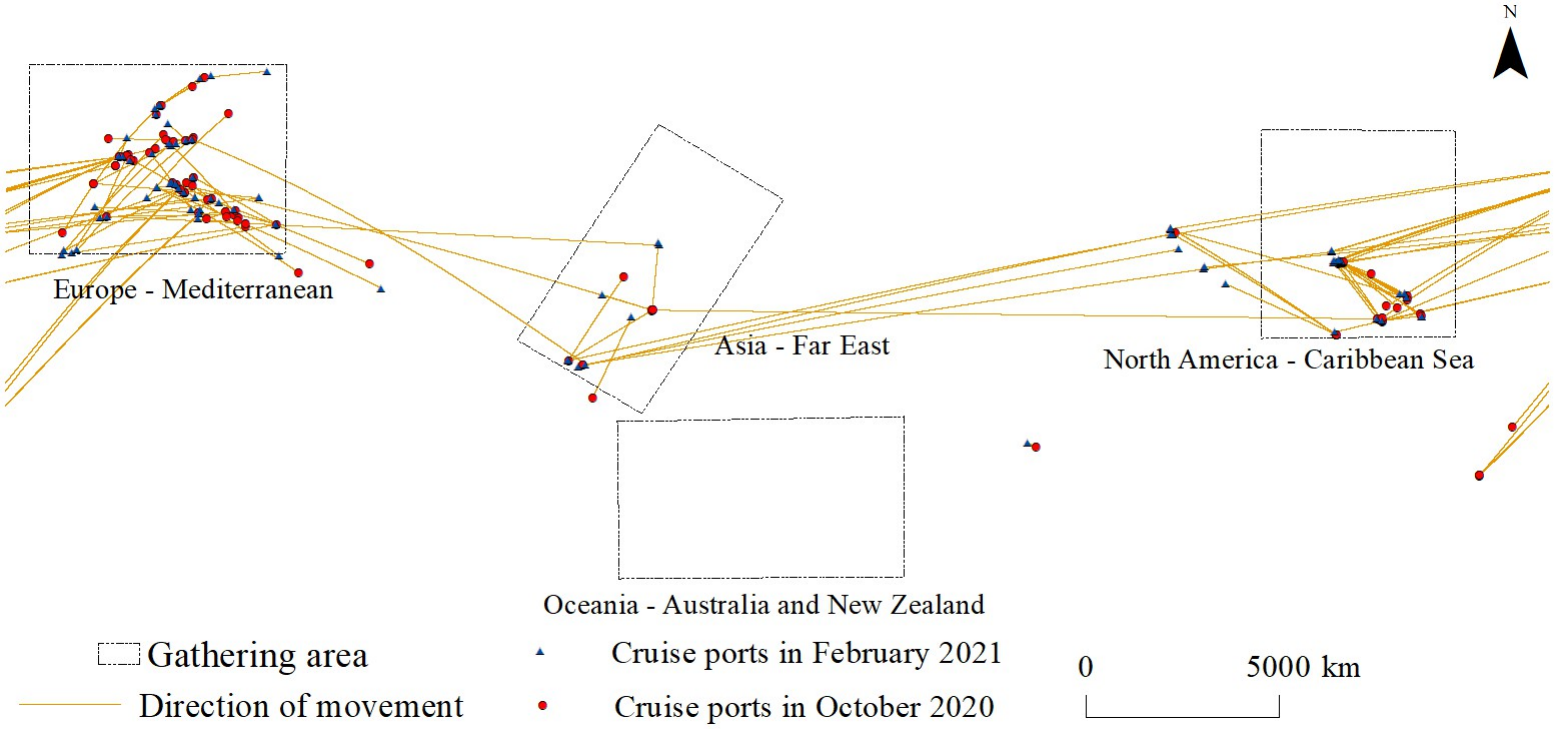
Spatial displacement of global cruise ships during the COVID-19 pandemic.

By comparing the spatial positions of cruise ships at different points during the pandemic, the study finds that not all cruise ships are relatively stationary. Comparing the two-time nodes from March 2020 to July 2021, 36.3% of cruise ships experienced significant spatial displacement (excluding those that moved irregularly in the waters near ports). Twenty-eight operators, including AIDA Cruises, Carnival Cruise Line, and Celebrity Cruises, had more than 10 ships in their fleets, accounting for 39.3%, and 26 operators had more than 20% of their ships moving. Eleven operators, including TUI Cruises, Holland America, and Hapag-Lloyd Cruises Fleet, had more than 50% of their ships moving. Among them, 80% of ships of TUI Cruises, Holland America, and Hapag-Lloyd Cruises Fleet had moved (Fig 7).

From the perspective of regional spatial variation, In the Region of the Americas, some cruise ships distributed in the South Caribbean Region, the East Caribbean region, Western Europe, and the Mediterranean region in 2020, and they migrated to the Florida Peninsula, the west coast of North America and the waters near the Bahamas in 2021, and the ports of call were mainly Miami port, Canaveral port, and Los Angeles Port. Cruise ships from Santos port and El Salvador port in South America had moved to Delete port and Siracusa port in the Mediterranean. There was a two-way mutual migration between cruise ships in Europe and the Arabian Peninsula in West Asia, mainly involving Dubai port, Hurghada port, Genoa port, etc. Cruise ships in the eastern Mediterranean region moved more to ports in the western Mediterranean region. Cruise ships from western and northern Europe moved more to southwest Europe and the Mediterranean. In Asia, there was a two-way migration of cruise ships between China’s coastal areas and Southeast Asia. The main ports were Hong Kong port, Singapore port, and Mantra port. In addition, some cruise ships had moved from the port of Singapore to Western Europe.

From the perspective of the range of spatial displacement, only 20 cruise ships moved to different ports in the same country, and the country was mainly Italy and Norway. In terms of operators, all ships of Hurtigruten only moved between Norwegian ports, while all of the other 25 operators had moved across countries. For example, three cruise ships of Disney Cruise Line moved from Brest cruise port in France to Canaveral cruise port in the United States. Costa Cruises had five ships moving between Civitavecchia cruise port and La Spezia cruise port in Italy. seven cruise ships of MSC Cruises moved from Dubai cruise port, MSC Ocean Cay cruise port, Bahamas, Santos cruise port, Salvador de Bahia cruise port to Trieste, Italy cruise port, Genoa cruise port, etc. In addition, from the perspective of port functions, cruise ships of different operators had the characteristics of clustering to ports with the function of departure.

### Drivers of cruise migration

Until October 2020, the COVID-19 pandemic was in full outbreak and continued to spread around the world. The vast majority of coastal countries adopted strict epidemic prevention measures, and port entry and exit and cruise ship docking management were still controlled and prohibited. During this period, cruise ships mostly adopted the nearest port docking strategy. On the one hand, cruise operators held an optimistic attitude and predicted that cruise ships would resume sailing soon. On the other hand, it was also due to the consideration of the operation cost of the nearby docking of cruise ships.

After 2021, the global response to COVID-19 was normalized, and some countries lifted restrictions on cruise ship calls at ports. Based on the prediction of the recovery time, operators comprehensively compared cruise maintenance, cruise schedule seasonality, peak season and low season in regional markets, etc., and rescheduled cruise ships worldwide to ensure minimum operating costs and to resume sailing as far as possible in conditions for resumption and market areas within peak season. This prompted more cruise ships to migrate to ports with cruise ship departure functions.

## State policy, the relationship between port and cruise line

The study finds that the spatial agglomeration of the global cruise shipping network in normal times is similar to that during the COVID-19 pandemic, which is affected by multiple factors such as the tourist market, regional shipping conditions, tourism resources richness, and navigation culture. However, the epidemic control policies of countries taking measures are the primary influencing factor for the cruise ship agglomeration pattern under the influence of the epidemic, which determines which countries cruise ships can dock in. What this reflects is the problem of the country attribute of a cruise, including (flag state, operator’s country, and passenger’s country). In coastal countries where cruise ships are allowed to dock, which ports cruise ships dock at actually shows the relationship between operators and ports. The relationship may be that the operator owns the port, or the operator owns the right to use the port.

### The contradiction between country and cruises under COVID-19

During the COVID-19 pandemic, most countries have imposed travel bans to prevent the virus from spreading globally via tourist flow. To prevent the importation of viruses at port nodes, coastal countries have adopted stricter port management policies for the entry and exit of people. In the early days of the COVID-19 pandemic, both passenger and non-passenger cruise ships were rejected or restricted by some countries. From the point of view of state relations, this mainly lies in the differences between port states, ship countries, and operator countries.

1. The relationship between the country where the port is located and the country where the ship is registered. For cruise ships, the owner of the ship registers the ownership of the ship with the relevant domestic or foreign administrative department in charge of the ship, to obtain the nationality of the country or the registration country, and the flag of the ship is to show the ownership of the registration country of the ship. For the cruise port, the operation and management of the port should follow the relevant laws and regulations of the owning country. Statistics show that cruise registration countries are mostly small countries or developed countries in Europe and the United States, which mostly adopt open registration systems or second ship registration systems. According to statistics, cruise ship registration countries are mostly small countries or developed countries in Europe and the United States, which mostly adopt open ship registration systems or second ship registration systems, including Bahamas, Malta, Panama, Norway, Italy, Germany, the United Kingdom, France, Portugal, etc. These countries account for nearly 70% of all cruise ships at port. This shows that under the influence of COVID-19, the spatial location of cruise ships is closely related to their nationality.
2. The nationality of the operator refers to the country to which the cruise operator belongs, and its production and operation activities are subject to the laws of that country. There are two main types of cruise operator nationalities, which are the incorporation rules and the main seat of business rules. Cruise operators are mainly registered in the United States, the United Kingdom, Norway, Germany, Greece, France, and Malta. During the COVID-19 pandemic, 87.5% of cruise ships docked at ports in these countries. The location of an operator’s headquarters can reflect its main place of business. Cruise operators are mainly based in the United States, the United Kingdom, Germany, Italy, France, Greece, and Norway. More than 83% of cruise ships stop at ports in these countries. At the same time, the cruise operator’s place of registration and place of business is usually a country. These countries are mainly concentrated in the United States, the United Kingdom, Norway, Germany, Greece, France, Italy, and other countries in Europe and America, which is strongly correlated with the spatial distribution pattern of cruise ships during the COVID-19 pandemic.

### The power relationship between cruise lines and ports

The relationship between port and navigation is usually manifested as the relationship between port enterprises and cruise operators. Cruise operators and port enterprises have an interdependent symbiotic relationship, which can be summarized as a mutual benefit based on both sides [35].

Generally speaking, the degree of port privatization in European and American countries is higher. No matter whether large cruise operators take equity shares in port terminals or acquire the ownership of terminals completely, or if port enterprises acquire cruise operators, the degree of port and navigation integration can be improved. Cruise ports with a high degree of port and navigation integration docked more cruise ships when cruise berthing restrictions were imposed due to the COVID-19 pandemic. In addition, some large cruise operators have ownership of individual islands and built their special port terminals, which also forms the phenomenon of island ports for cruise ships to berth. Large cruise operators, such as Royal Caribbean, Carnival, and Norwegian cruise lines, dock some of their cruise ships at Caribbean island ports, especially the Bahamas and Barbados. Moreover, the region is also an overseas territory of the United Kingdom, the United States, France, the Netherlands, and other countries, including Puerto Rico, the Virgin Islands, Anguilla, St. Martin, etc., which forms the spatial pattern of cruise agglomeration in the eastern Caribbean island region.

## Conclusions and discussion

### Conclusions

The global spread of COVID-19, while having a serious impact on the cruise shipping industry, has also provided us with a special opportunity to study the spatial distribution of cruise ships in relative immobility. This will expand the content of the global cruise shipping network and help us to understand the geospatial characteristics and laws of the cruise shipping network in a special period more comprehensively.

By studying the spatial distribution pattern and movement path of global cruise ships under the influence of COVID-19, it is found that the spatial aggregation characteristics of cruise ships have strong regional similarities compared with normal periods. This similarity is embodied in the agglomeration of cruise ships in major global regional markets such as North America and Europe, as well as in major sea areas such as the Caribbean Sea, the Mediterranean Sea, and the Baltic Sea. The difference is that the distribution of cruise ships has a significant spatial contraction at the country and port levels. More cruise ships tend to gather in Italy, the United States, Greece, and other countries. From the perspective of the port, more cruise ships choose to cluster in port areas with the function of departure, especially Miami port, Singapore port, Piraeus port, and other cruise home ports. As the direct organizer of cruise ships, cruise operators’ deployment of different cruise ships with operation quantity and route positioning shows the characteristics of a single regional agglomeration, two regional agglomerations, and a multi-regional agglomeration. With the expansion of fleet size, operators will gradually increase the deployment area for cruise ships. However, large cruise operators such as Carnival Cruise Line and Celebrity Cruises will also concentrate a considerable number of cruise ships in one or two major cruise home ports. With the easing of COVID-19 and the change in national policies, cruise operators have adjusted their expectations for the resumption of flights promptly and organized cruises to migrate between different regions and ports according to seasonal changes in regional markets. The number of cruise ships that migrate accounts for more than a third of the total cruises.

### Discussion

We believe that the COVID-19 pandemic forces a country to elevate its health security to a high level, even above its economic development. In this context, different countries will choose to exercise their rights to prevent the import of the epidemic through cruise ships. In this process, the port epidemic prevention countries, cruise flag states, cruise operators, and other multi-stakeholder parties will form "rights factors" at different levels such as port state sovereignty, port use rights, port management rights, cruise ownership, cruise management rights, etc. The opposition and unity between them have become the key to determining the spatial distribution of the global cruise shipping network.

Therefore, from the perspective of the healthy development of the cruise shipping network, the first thing is to ensure the health and safety of the cruise ship operation. On this basis, it is crucial to strengthen the cooperation between countries and between port companies and shipping companies through regional cooperation, strategic alliances, multinational enterprises, joint ventures, integration, and other diversified forms. This can avoid the extreme situation where one stakeholder is dominant or two stakeholders are completely isolated from each other and thus improve the robustness of the global cruise shipping network in special situations and periods such as health security.

This study focuses on the static pattern of cruise networks, while current studies pay more attention to the network characteristics and rules under the dynamic changes of cruise ships. The static cruise network under COVID-19 is essentially a microcosm of the cruise network in normal times, especially at the port scale. Therefore, this study reveals the other side of the cruise network, which contributes to a more comprehensive understanding of the formation, development, and internal dynamics of the global cruise network. Meanwhile, it should be noted that this study only selects the key time node data under the influence of the new coronavirus as the research sample, which is selected after comparing the data of different time nodes. Therefore, this part of the data is highly representative and can objectively reveal the internal law. However, inevitably, the limited node data cannot fully reflect the changes in port docking distribution and movement path of some cruise ships outside these time nodes, which becomes a flaw in this study.

## Supporting information

S1 Data (XLS)
